# Validation of Self-Reported Smoking Status Among Orthopedic Hip Surgery Patients

**DOI:** 10.7759/cureus.10753

**Published:** 2020-10-01

**Authors:** Samuel T Ellis, Brian M Rao, Dave Kohlrieser, Robert C Kollmorgen, Kyle R Sochacki

**Affiliations:** 1 Orthopedics, Orthopedic One, Dublin, USA; 2 Hip Preservation and Sports Medicine, University of California San Francisco, San Francisco, USA; 3 Orthopedics and Sports Medicine, Houston Methodist Hospital, Houston, USA

**Keywords:** hip, arthroscopy, peri acetabular osteotomy, arthroplasty, smoking, cessation, nicotine, cotinine, orthopedic, surgery

## Abstract

Purpose

The purpose of this study was to determine the accuracy of self-reported non-smoking status in subjects undergoing elective orthopedic surgery as confirmed by serum cotinine levels.

Methods

Institutional Review Board approval was obtained for this retrospective review of consecutive subjects that underwent elective orthopedic surgery by a single fellowship-trained orthopedic surgeon. All patients provided smoking history (active, former, or non-smoker). Serum cotinine levels defined each subject as “non-smoker”, “passive tobacco exposure”, or “active smoker”. Self-reported non-smokers were eligible for inclusion. Subjects were excluded if they failed to provide smoking history, reported themselves as “smokers”, and/or had unavailable serum cotinine levels. Self-reported non-smoking status accuracy was determined by dividing the total number of included subjects by the number of subjects that were defined as “non-smoker” or “passive tobacco exposure” on their serum cotinine test.

Results

A total of 378 patients (mean age of 42.5 (13-78) years and 68% female) self-reported as non-smokers and were included. A total of 369 subjects had serum cotinine levels consistent with “non-smoking” resulting in a self-reported non-smoking status accuracy of 97.6%. None of the former smokers had cotinine levels consistent with active smoker status.

Conclusion

Subjects undergoing elective orthopedic surgery self-report as non-smokers with an accuracy of 97.6%. This suggests that routine serum cotinine testing of non-smokers in this patient population may not be necessary.

## Introduction

Tobacco use in the United States (U.S.) remains problematic, despite a long-term decline [1]. According to the Centers for Disease Control and Prevention (CDC), cigarette smoking is the leading cause of preventable death in the U.S. as it is responsible for more than 480,000 deaths each year [2]. Compared to non-smokers, smokers are more likely to develop cancers, cardiovascular disease, pulmonary disease, impaired immune function, and reduced life expectancy [3].

Smokers are also at a higher risk for surgical and postoperative complications [4-6]. Previous studies have demonstrated that smoking increases the risk for wound complications and leads to nearly double the number of surgical site infections [7,8]. The same trend has also been seen in orthopedic trauma patients with open fractures. Smokers were twice as likely to develop an infection, 3.7 times more likely to develop osteomyelitis, and twice as likely to experience non-union after osteotomy [9,10]. These complications can be greatly reduced by at least 40% if smoking cessation is completed at least four weeks prior to surgery [11].

Historically, routine nicotine and cotinine screening of patients prior to surgery has been performed. Nicotine remains detectable in the blood up to three days after exposure, while cotinine, the primary metabolite of nicotine, is detectable up to 10-14 days in urine, saliva, and blood after exposure [12-14]. However, as increased scrutiny is placed on reducing perioperative surgical complications, modifiable risk factors including smoking status have been looked at more critically in recent years [15,16].

This has resulted in clinical care pathways to identify prior and current smoking and urge cessation at each visit. Many physicians routinely inquire about patient smoking history on medical history forms provided at their initial clinic visit. Previous studies have raised concerns about the validity of patients self-reporting smoking status, as some smokers may report as non-smokers [17-20]. However, there is limited data on the veracity self-reported smoking status among patients undergoing elective orthopedic surgery. The purpose of this study was to determine the accuracy of self-reported non-smoking status in subjects undergoing elective orthopedic surgery as confirmed by serum cotinine levels. The authors hypothesized that there would be greater than 90% accuracy of self-reported non-smoking status.

## Materials and methods

Mount Carmel Institutional Review Board (IRB Protocol #: 190509-3) approval was obtained for this retrospective study of consecutive subjects that underwent elective hip surgery by a single fellowship-trained orthopedic surgeon between July 2016 and June 2017. All patients completed a General Health Questionnaire that included current symptoms, treatments, past medical history, medications, allergies, review of systems, and social habits including smoking history (active, former, or non-smoker) or tobacco user at their initial clinic visit and annually thereafter. The questionnaire was mailed to patients prior to their initial appointment and completed prior to seeing the physician. All forms were completed exclusively by the patient without assistance from any staff or research personnel (research assistants, fellows, residents, students, nurses, physicians, physician assistants, or nurse practitioners). Once completed, the forms were scanned into the electronic medical record (EMR). Subjects were not informed that they would be undergoing serum cotinine testing prior to completing the questionnaire.

Venous blood samples for serum cotinine levels from all surgical patients were taken prior to surgery as standard practice. According to prior studies, serum cotinine levels were used to define each subject as “non-smoker”, “passive tobacco exposure”, or “active smoker” (Table 1) [12,21].

**Table 1:** Non-smoker, passive tobacco exposure, or active smoker values. Values expressed in nanograms (ng) per milliliter (ml).

Tobacco Exposure	Serum Cotinine Level (ng/ml)
Non-smoker	<3
Passive tobacco exposure	3-8
Active tobacco use	>8

All subjects that reported as “non-smokers” on their questionnaire were eligible for study inclusion. Subjects were excluded if they did not complete the smoking portion of the questionnaire; reported themselves as “smokers", "vapors", and "smokeless tobacco users"; and/or had serum cotinine levels that were unavailable or never drawn.

Demographic information such as age, sex, ethnicity, surgical procedure, insurance, serum cotinine level, and current/former smoking status was recorded for each subject. Continuous variable data were reported as mean and range. Categorical variable data were reported as frequency with percentage. Self-reported non-smoking status accuracy was determined by dividing the total number of included subjects by the number of subjects that were defined as “non-smoker” or “passive tobacco exposure” on their serum cotinine test.

## Results

**Figure 1:**
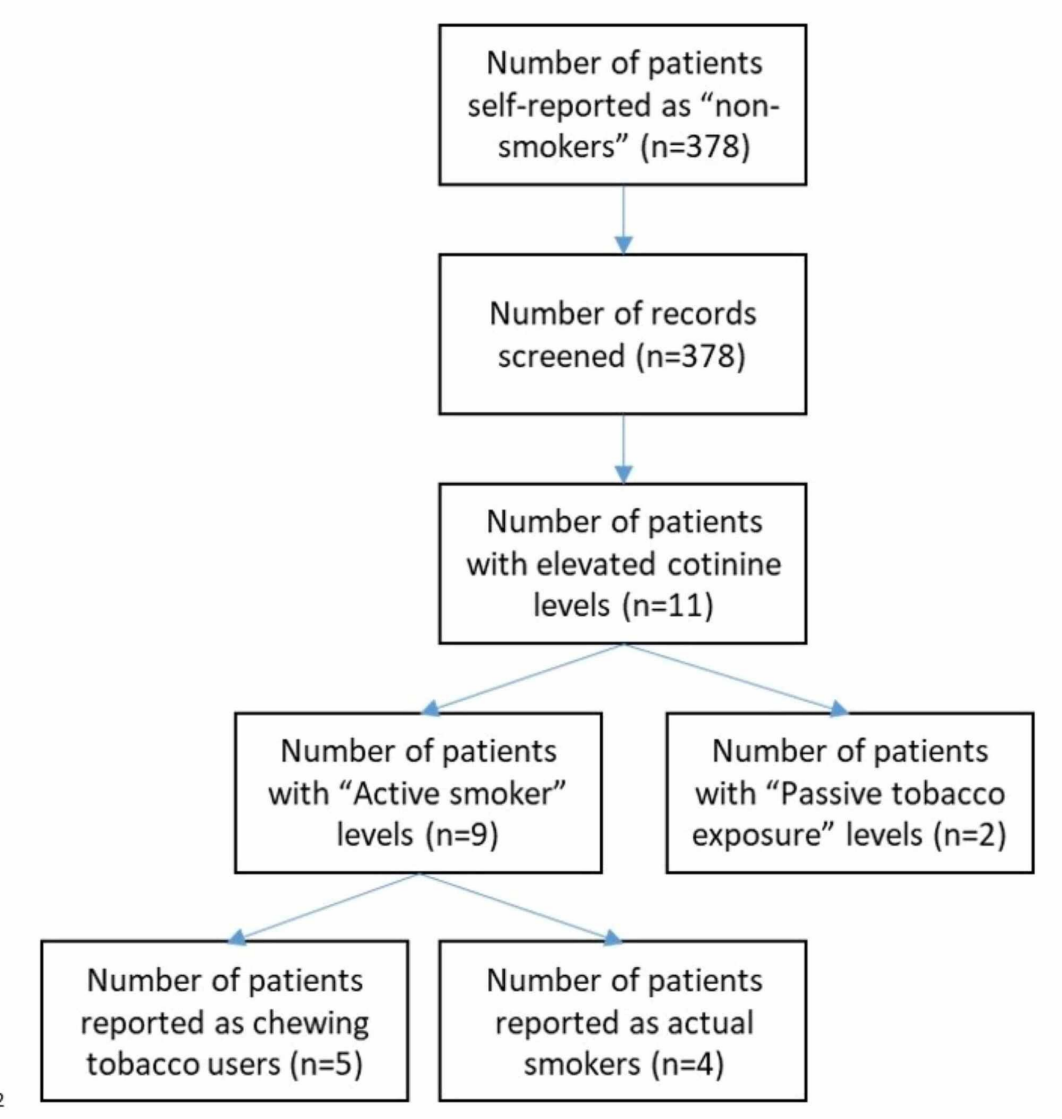
Flow diagram of patient smoking statuses

A total of 378 patients (mean age of 42.5 (13-78) years and 68% female) self-reported as non-smokers and were included in the study (Figure 1, Table 2). Thirty subjects were former smokers. The majority of patients were white, 332 (87.8%) subjects had commercial insurance, and 31 (8.2%) were insured by Medicare (Table 2). The most commonly performed procedures were total hip arthroplasty (51.1%), hip arthroscopy (31.7%), and periacetabular osteotomy (11.1%) (Table 3).

**Table 2:** Patient composition

Variables		Count (n)	Percentage (%)
Age	<18 years	28	7.4
	18-30 years	80	21.2
	31-40 years	57	15.1
	41-50 years	75	19.8
	51-60 years	73	19.3
	61-70 years	51	13.5
	>70 years	14	3.7
Sex	Male	120	32
	Female	258	68
Race	White	344	91
	Black	7	1.9
	American Indian or Alaska Native	5	1.3
	Asian	3	0.8
	Hispanic	3	0.8
	Unreported	16	4.2
Insurance	Commercial	332	87.8
	Medicare	31	8.2
	Military	9	2.4
	Workers Compensation	5	1.3
	Self-pay	1	0.3

**Table 3:** Types of surgery PAO, periacetabular osteotomy; HO, heterotopic ossification

Type of Surgery	Count (n)	Percentage (%)
Total hip arthroplasty	193	51.1
Hip arthroscopy	120	31.7
PAO	42	11.1
Gluteus medius repair	8	2.1
Tendon lengthening	7	1.9
Femoral osteotomy	5	1.3
Removal hardware	2	0.5
Excision of HO	1	0.3

Eleven (2.9%) subjects had elevated serum cotinine levels (Table 4). Nine (2.4%) of these subjects had levels that were consistent with “active smoker” and two (0.5%) subjects had elevated cotinine levels consistent with “second-hand tobacco exposure”. A total of 369 subjects had serum cotinine levels that were consistent with “non-smoking” resulting in a self-reported non-smoking status accuracy of 97.6%. Of the 378 subjects, 5 confirmed used chewing tobacco as opposed to smoking leading to their elevated serum cotinine. Only four (1.1%) subjects that initially self-reported non-smoking had elevated serum cotinine levels consistent with “active smokers” and later confirmed active smoking status. One of the self-reported former smokers had elevated cotinine levels consistent with second-hand smoke exposure. This patient reported their spouse was a smoker. None of the former smokers had cotinine levels consistent with active smoker status.

**Table 4:** Blood test results

	Count (n)	Percentage (%)
Non-smoker	367	97.1
Passive tobacco exposure	2	0.5
Active smoker	9	2.4

## Discussion

This study aimed to assess the accuracy of self-reported non-smoking status in subjects undergoing elective orthopedic surgery. It was determined that the accuracy of self-reported non-smoking status on routine intake forms was 97.6%. This study confirms the authors’ hypothesis.

Smoking has been shown to increase the risk of perioperative complications, including infection, wound complications, and delayed or non-union [4-10]. As such, clinical care pathways to identify prior and current smoking and urge cessation at each visit have been established [22]. Many physicians routinely inquire about patient smoking history on medical history forms provided at their initial clinic visit with Wong et al., concluding that accurate estimates of the prevalence of cigarette smoking can be derived from self-reported smoking status [20]. 

However, comparing self-reported smoking status with preoperative serum or urine cotinine levels has raised some concerns about the reliability of self-reporting [12,17-20]. Several recent studies have demonstrated that self-reported smoking status underestimates the true smoking incidence by up 45% [17-20,23-25]. This is significantly worse than the present study where 97.6% of subjects accurately reported as non-smokers, as confirmed by negative serum cotinine levels.

There are several explanations for this discrepancy. The current study population is from a large community-based orthopedic practice in a metropolitan setting that is predominantly white (91.0%) with commercial insurance (87.8%) and a mean age of 42.5 years. This is very different from the typical patients seen in an urban-based academic medical center and likely plays a large role in the accuracy of self-reported smoking status. Arheart et al. found that minority populations self-report their smoking status with significantly less accuracy compared to non-minority patients [25]. Additionally, prior studies have shown that younger patients (<19 years) and those with poorer socioeconomic status are less likely to accurately report on their smoking status compared to their older and more affluent counterparts seen in the present study [20,23].

The subspecialty clinics that the patients present to also likely plays a major role in the accuracy of self-reported smoking. Of those previously studied, pregnant patients and those undergoing surgery for head and neck cancer were the least accurate in their reporting of smoking [17,19,23,24]. There is likely a higher stigma associated with smoking during pregnancy and with head and neck cancer compared to elective orthopedic surgery. This is further illustrated with patients undergoing orthopedic surgery demonstrating self-reported smoking accuracy that ranges from 85% to 96.3% in previous studies for non-smokers [12,18,26]. This is much more similar to the results of the present study (97.6% accuracy of self-reported smoking status).

There are a few differences, however, between the current and previous studies in orthopedic patients. The previous studies found that former smokers were significantly less accurate with their self-reporting compared to those who never smoked [12,17,18]. This compares to no former smokers having elevated cotinine levels consistent with active smoker status in the present study. One subject did have elevated cotinine levels from second-hand smoke exposure by their spouse. The prior studies in orthopedic surgery patients include a large proportion undergoing trauma surgery [17,18]. These patient populations are often very different than those undergoing elective surgery and may have contributed to their slightly reduced accuracy of self-reporting. Additionally, subjects in the current study were informed after the questionnaire was completed that their surgery would be cancelled if cotinine levels were consistent with an “active smoker”. Hart et al. recently determined that knowledge of preoperative cotinine testing increases smoking cessation preoperatively as measured by serum cotinine [12]. This likely led to an inflated accuracy of self-reporting compared to the present study.

The goal of preoperative smoking cessation is to reduce the risk of perioperative and postoperative complications [27]. This is especially important when considering revision total hip arthroplasty that has been estimated to cost $77,851.24 per case [28]. This raises the question of the cost-effectiveness of routine serum cotinine testing. Salandy et al. cited a cost of £7.00 and £1.50 for serum and urine cotinine testing, respectively [26]. Given this difference in cost, the authors concluded that serum cotinine testing should only be reserved in high-risk patients who have elevated urine cotinine levels. This is especially true based on the results of the present study with 97.6% accuracy of self-reported non-smoking. As such, it is the current authors' opinion that preoperative serum cotinine testing may be more applicable in patients who report as active or former smokers to ensure compliance with smoking cessation prior to surgery. However, a formal cost-benefit analysis is beyond the scope of the present study and should be a topic of future research in order to determine the number needed to test in order to reduce a single revision surgery.

It is also important to discuss the role of smokeless tobacco and vaping in preoperative patients. However, to a lesser extent compared to smoking, smokeless tobacco has been shown to increase the risk of mortality and cardiovascular disease [29]. Vaping is an emerging problem and is associated with increased perioperative complications [30]. Most of the current literature has focused on a patient’s smoking history as opposed to their tobacco history which may affect the accuracy of their self-reporting. The present study is no different with all subjects provided a questionnaire inquiring about smoking history (active, former, or non-smoker). This led to a self-reporting non-smoking accuracy of 97.6% with nine subjects having serum cotinine levels consistent with active smoking. However, upon further analysis, five of these subjects reported smokeless tobacco and only four were truly “active smokers”. In addition, then questionnaire did not ask specifically about "vaping". As such, the authors recommend additional screening for smokeless tobacco or vaping history in order to properly risk stratify and counsel patients about the benefits of all tobacco/vaping cessation to reduce the risk of postoperative complications.

There are some limitations to this study. The study was retrospective possibly leading to selection bias. All subjects were patients of a single fellowship-trained orthopedic surgeon in a large community-based orthopedic practice in a metropolitan setting that is predominantly white with commercial insurance. Thus, the results of this investigation may not be extrapolated to all patient populations. Additionally, serum cotinine levels were used as the gold standard against which self-reported smoking history was compared. Serum cotinine testing is not 100% sensitive or specific for detecting smoking status, so it is possible that some active smokers were not detected. However, the values used in the present study have been validated in previous studies [12,21]. Only subjects that self-reported as non-smokers were included in the study limiting a comparison between the accuracy of active and non-smoking self-reporting. As such, a reduction in postoperative complications through serum cotinine testing was unable to be determined in the present study.

## Conclusions

Subjects undergoing elective orthopedic surgery self-report as non-smokers with an accuracy of 97.6%. This suggests that routine serum cotinine testing of non-smokers in this patient population may not be necessary.
